# Delayed Pneumothorax Post Transbronchial Biopsy: A Case Report

**DOI:** 10.7759/cureus.14614

**Published:** 2021-04-21

**Authors:** Mohammed Alsaggaf, Ali Khalofa, Rahul Khosla

**Affiliations:** 1 Pulmonary and Critical Care Medicine, George Washington University, Washington, DC, USA; 2 Pulmonary and Critical Care Medicine, Veterans Affairs Medical Center, Washington, DC, USA

**Keywords:** trans bronchial biopsy, pneumothorax (ptx), secondary pneumothorax, risk factors for pneumothorax, presentation delay

## Abstract

Bronchoscopy is a common and safe procedure with low mortality rates and complications. The risk of pneumothorax (PTX) post bronchoscopy is estimated to be 0.1% but increases to 1-6% with the addition of transbronchial lung biopsy (TBB) to the procedure. Studies have shown that a short observation period is adequate after TBB, and the usual practice is to perform a portable chest radiograph (CXR) to rule out PTX. Delayed PTX is a rare complication post-TBB and very few cases have been reported in the literature. In this report, we discuss a patient with delayed PTX 48 hours post-TBB.

A 71-year-old male with a history of malignancy of unknown primary with metastasis to the sacrum and vertebral column presented with lower limb weakness status post-palliative radiation to the spine. His sacral lesion biopsy was inconclusive. He was currently on oral steroids. He was noted to have a left upper lobe lung nodule on a CT scan of the chest. He underwent bronchoscopy with TBB to determine if it was a primary lung malignancy. He was stable post-procedure with an unremarkable CXR for PTX and was discharged with outpatient follow-up. Two days later, he presented to the emergency department with shortness of breath and hypoxemia. A CXR was performed, which showed a left-sided PTX. A chest tube was placed, and a follow-up CXR showed lung immediate re-expansion. The chest tube was removed after two days and the patient was discharged home after a total of four days of hospitalization.

Iatrogenic PTX can be due to diagnostic and/or therapeutic interventions. PTX after procedures can be classified as acute (one to four hours post-procedure) or delayed (>4 hours post-procedure). It is recommended to have a CXR within an hour post-TBB. To our knowledge, very few cases of delayed PTX post-TBB have been reported, mostly among lung transplant patients and those with chronic infections such as tuberculosis. In prior reports, it has been speculated that a visceral pleural defect might occur during a biopsy, but is protected by blood clot formation in the proximal bronchus. A PTX then occurs after fibrinolysis of the blood clot. Low immunity and poor wound healing due to chronic inflammation or steroid use can play a role in causing a delayed PTX. Also, the use of pain drugs such as opioids is associated with iatrogenic PTX. Patients with underlying lung disease such as emphysema are more prone to developing a PTX. Another hypothesis is that a tissue flap is created after the biopsy, which obstructs the airflow during exhalation, thereby resulting in a PTX. On the other hand, it is known that lung malignancies, either primary or metastatic, can increase the risk of secondary PTX. In our case, the temporal relationship of the delayed PTX with bronchoscopy makes it more likely that it was related to the lung biopsy (in our case, poorly differentiated non-small cell carcinoma). The underlying malignancy with low immunity, chronic tissue inflammation, and current steroid use may have resulted in delayed lung healing at the biopsy site. This case report highlights the importance of considering delayed PTX among high-risk patients who undergo such procedures.

Delayed PTX is a rare complication post-TBB and should be considered in patients who are stable post-procedure but present with dyspnea and/or hypoxemia even days after the procedure.

## Introduction

Bronchoscopy is a common medical procedure. It is considered very safe and is associated with low mortality rates and complications. The risk of patients developing pneumothorax (PTX) after this procedure is very low and is estimated to be at 0.1%. However, it increases to 1-6% [1]^ ^with the addition of transbronchial lung biopsy (TBB) to the procedure. As per published literature, a short observation period is adequate after TBB, and a portable chest radiograph (CXR) is usually performed to rule out PTX [2]. Delayed PTX is a rare complication post-TBB and very few cases have been reported in the literature [3]. In this report, we present the case of a patient with delayed PTX 48 hours post-TBB.

## Case presentation

**Figure 1:**
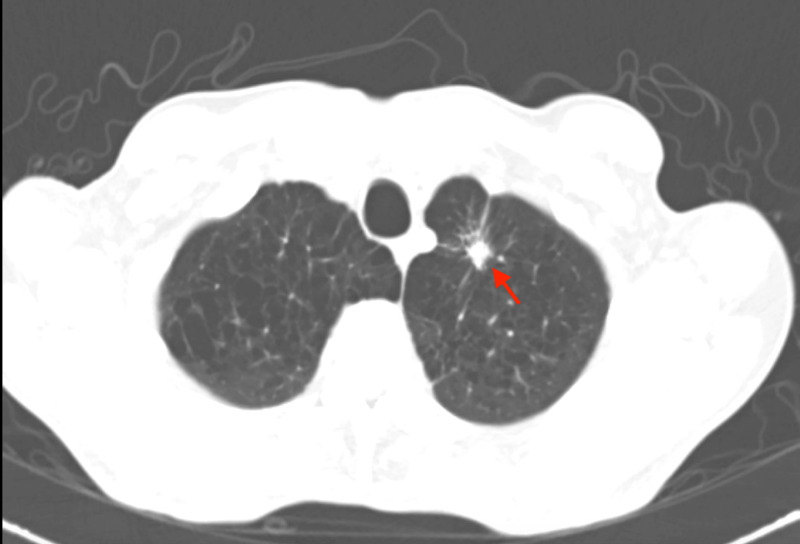
CT chest showing left upper lobe lesion (arrow) CT: computed tomography

**Figure 2:**
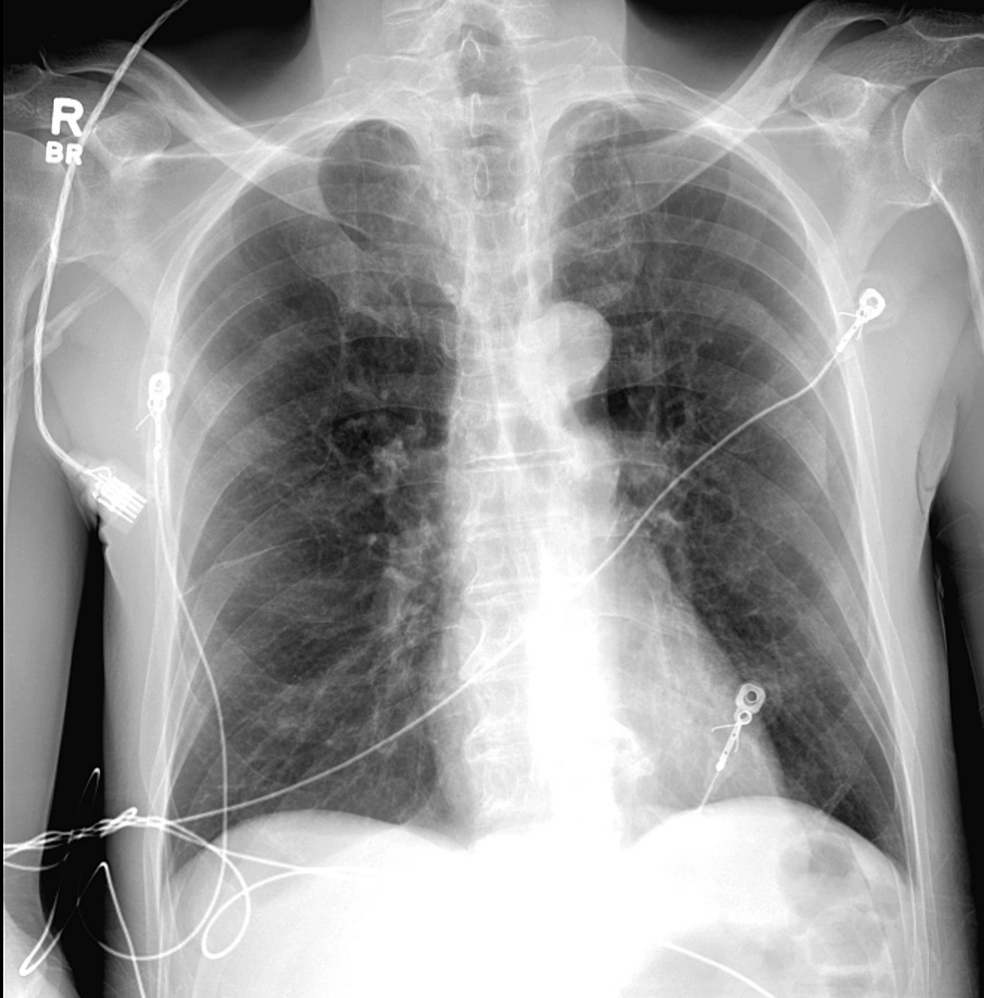
CXR post-biopsy without PTX CXR: chest radiograph; PTX: pneumothorax

**Figure 3:**
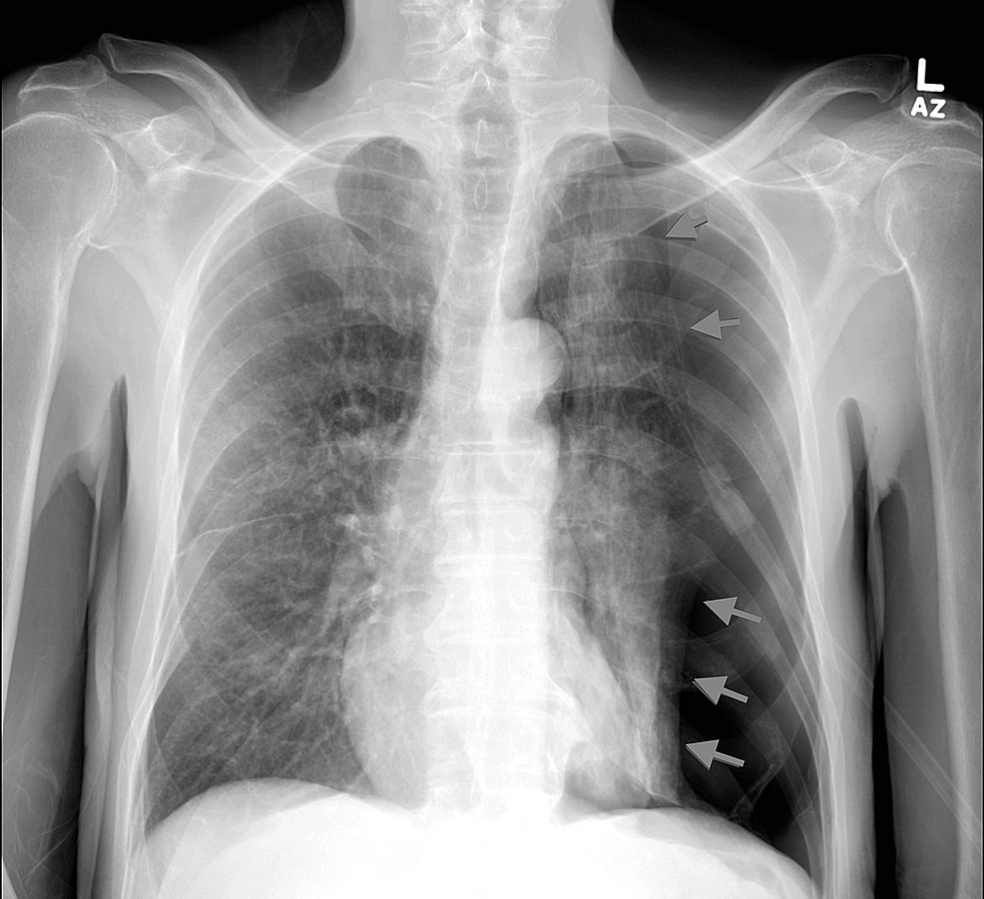
CXR with delayed PTX post-biopsy (arrows) CXR: chest radiograph; PTX: pneumothorax

Our patient was a 71-year-old male, with a medical history remarkable for malignancy of unknown primary with metastasis to the sacrum and vertebral column, who presented with lower limb weakness status post-palliative radiation to the spine. His sacral lesion biopsy was found to be inconclusive. He was currently on oral steroids. A CT scan of the chest revealed a left upper lobe lung nodule (Figure 1). A bronchoscopy with TBB was performed to determine if it was a primary lung malignancy. The patient was stable post-procedure, and the CXR for PTX was unremarkable (Figure 2). He was subsequently discharged with instructions for outpatient follow-up. Two days later, he presented to the emergency department complaining of shortness of breath and hypoxemia. The CXR showed a left-sided PTX (Figure 3). A chest tube was placed, and a follow-up CXR showed lung immediate re-expansion. The chest tube was removed after two days and the patient was discharged home. He had spent a total of four days in the hospital.

## Discussion

Iatrogenic PTX can result from diagnostic and/or therapeutic interventions. This kind of post-procedure PTX can be classified into two types: acute (one to four hours post-procedure) or delayed (>4 hours post-procedure) [2]. It is ideal for the patient to undergo a CXR within an hour post-TBB [2]. To the best of our knowledge, very few cases of delayed PTX post-TBB have been reported [3]; and these incidents have been found mostly among lung transplant patients and those suffering from chronic infections such as tuberculosis [3-4]. It has been hypothesized in previous studies that a visceral pleural defect might occur during the biopsy, but is protected by blood clot formation in the proximal bronchus. A PTX then occurs after fibrinolysis of the blood clot. A delayed PTX can also result from low immunity and poor wound healing due to chronic inflammation or steroid use. Additionally, iatrogenic PTX has also been associated with the use of pain medications such as opioids. The presence of underlying lung diseases such as emphysema can make patients more susceptible to developing a PTX [5]. According to another hypothesis, a tissue flap is created after the biopsy, which obstructs the airflow during exhalation, which consequently leads to PTX. On the other hand, lung malignancies, either primary or metastatic, are known to increase the risk of secondary PTX [5]. In our patient, the temporal relationship of the delayed PTX with bronchoscopy makes it more likely that it was related to the lung biopsy (in our case, poorly differentiated non-small cell carcinoma). The underlying malignancy coupled with low immunity, chronic tissue inflammation, and current steroid use may have led to delayed lung healing at the biopsy site. This case report highlights the importance of considering delayed PTX as a diagnosis among high-risk patients who undergo such procedures [6]. Table 1 lists the various causes of PTX.

**Table 1:** Causes of pneumothorax TPP: transpulmonary pressure; COPD: chronic obstructive pulmonary disease; CF: cystic fibrosis; TB: tuberculosis; PJP: pneumocystis jiroveci pneumonia; CTD: connective tissue disease; EDS: Ehlers-Danlos syndrome

Spontaneous		Traumatic	
Primary (no lung disease)	Secondary (lung disease)	Iatrogenic	Non-iatrogenic
Tall, thin, and smoker	Airway: COPD, CF, asthma	Transbronchial biopsy (TBB)	Penetrating chest wall injury
High TPP: diving	Infection: TB, PJP	Procedure: central line/thoracentesis	Blunt force trauma -/+ rib fracture
	CTD	Mechanical ventilation	
	Interstitial lung disease	Pacemaker insertion	
	Malignancy	Thoracic surgery	

## Conclusions

We discussed the case of a 71-year-old male who was treated successfully for delayed PTX that he had developed after undergoing TBB. Delayed PTX is a rare complication post-TBB and should be considered in patients who are stable post-procedure but present with dyspnea and/or hypoxemia even days after the procedure.
